# The Relations and Role of Social Competencies and Belonging with Math and Science Interest and Efficacy for Adolescents in Informal STEM Programs

**DOI:** 10.1007/s10964-020-01302-1

**Published:** 2020-08-17

**Authors:** Adam J. Hoffman, Luke McGuire, Adam Rutland, Adam Hartstone-Rose, Matthew J. Irvin, Mark Winterbottom, Frances Balkwill, Grace E. Fields, Kelly Lynn Mulvey

**Affiliations:** 1 Western Carolina University, Cullowhee, NC USA; 2 University of Exeter, Exeter, UK; 3 North Carolina State University, Raleigh, NC USA; 4 University of South Carolina, Columbia, SC USA; 5 University of Cambridge, Cambridge, UK; 6 Barts Cancer Institute, Queen Mary University of London, London, UK; 7 Riverbanks Zoo and Garden, Columbia, SC USA

**Keywords:** Social competencies, Informal learning context, Belonging, Math and science efficacy, Math and science interest

## Abstract

Adolescence represents a developmental period of waning academic motivation, particularly in STEM domains. To combat this, better understanding the factors that might foster STEM motivation and interest is of importance. Social factors like social competencies and feelings of belonging become increasingly important in adolescence. The current study investigated structural relations between social competencies, feelings of belonging to an informal STEM learning program, math and science efficacy and interest in a sample of 268 adolescents (*M*_age_ = 15.25; 66.8% girls; 42.5% White British or European American, 25.7% South Asian British or Asian American, 15.7% Afro-Caribbean Black British or African American 5.6% Bi-racial, and 3.0% other). Adolescents were recruited from six different informal learning sites (e.g., science museums, zoos, or aquariums) in the United States (*n* = 147) and the United Kingdom (*n* = 121). The results revealed positive relations between social competencies and belonging, and between belonging and math and science efficacy and interest. Further, the results also indicated a positive indirect effect of social competencies on efficacy and interest, via belonging. These findings have implications for guiding informal STEM programming in ways that can enhance STEM motivation and interest.

## Introduction

Across adolescence, youth often experience decreased interest and efficacy in academic subjects (Wang and Eccles 2012), including in science, technology, engineering and mathematics (STEM) domains (Chen et al. 2016). If STEM motivation is not maintained during adolescence, it may be harder to improve STEM interests and related outcomes later in life. For instance, adolescents need to enroll in advanced courses in math and science in high school in order to be prepared for college and university STEM courses (Riegle-Crumb and King 2010). Research increasingly highlights the importance of attending to social-emotional development in understanding the overall academic trajectories of youth (DeRosier and Lloyd 2011; Domitrovich et al. 2017) and findings note that out-of-school settings are a particularly important context for fostering social-emotional development (Mahoney et al. 2005). The aim of the current study is to extend prior research on STEM interest and motivation in adolescence with attention to social factors in informal learning settings.

One such factor may be adolescents’ connections to and relationships with others, especially peers, which may hinge on adolescents’ social competence (Chen and French 2008). Social competence is “effectiveness in developmentally appropriate social interactions” (Denham et al. 2009, p. i38). It may be especially important for feeling that one belongs, particularly during adolescence when a sense of belonging becomes highly salient (Collins and Steinberg 2006). In a recent meta-analysis peers were identified as important agents in the lives of adolescents with regard to their feelings of belonging to their school (Allen et al. 2018). Further, the ability to effectively engage with peers and develop feelings of belonging are positively related to many academic and psychosocial outcomes (Durlak et al. 2011) and could play an important role in the maintenance and even the enhancement of STEM interest and motivation. Indeed, research has consistently shown that adolescents who have mastered more social skills compared to their peers report not only more effective learning strategies but also more development of interpersonal relationships, and greater sense of belonging (Wentzel et al. 2012). Interestingly, very little research has attended to the social factors of social competencies (e.g., ability to make new friends and get along with them) and feelings belonging to a larger organization (e.g., extracurricular program) and their structural relationship with STEM outcomes, particularly in learning contexts outside of the classroom. Thus, the present study fills this gap in the literature, as it assesses structural relations between social competencies, belonging, and STEM interest and efficacy in informal learning science sites.

### Theoretical Frameworks

Two theoretical frameworks jointly guided the hypotheses; namely, the social-emotional learning approach (Weissberg et al. 2015) and belongingness motivation theory (Baumeister and Leary 1995). The social-emotional learning approach posits that youth with enhanced skills in social and emotional competencies (i.e., who know themselves, take perspectives of others and relate to them, and make sound personal and social decisions) are likely to be more successful in both short- and long-term academic and psychosocial outcomes (Weissberg et al. 2015). Recent meta-analyses have documented the enduring effects of social-emotional learning for a wide-array of outcomes, highlighting that attention to social factors can promote positive youth development and academic success across contexts, including informal learning contexts (Durlak et al. 2011; Taylor et al. 2017). Youth with greater connections and sense of belonging can more efficaciously engage with peers and teachers (Durlak et al. 2011). Further, out-of-school, informal settings are key spaces where adolescents build connections with others and can develop feelings of belonging (Eccles et al. 2003; Mahoney et al. 2005).

The second theoretical framework is belongingness motivation theory (Baumeister and Leary 1995), which argues that belongingness is a critical social motive that underlies human cognition and behavior. Belonging is theorized to become more important as youth transition from childhood into adolescence, as youth become increasingly aware of the social world around them (Brown and Larson 2009). Research in formal school contexts has documented that school belonging is predictive of academic motivation over time (Gillen-O’Neel and Fuligni 2013). Less work, however, has attended to belonging in out-of-school settings, such as in informal learning programs. It may be that belonging is a key factor that explains how informal learning opportunities can promote STEM interest and motivation (e.g., self-efficacy).

### Social Competencies and Belonging

Youth who demonstrate better social skills (social competence) may have an easier time fitting in and finding their niche (belonging) within a particular setting. Empirical evidence has consistently provided support for this relationship in formal school settings. A recent meta-analysis that examined core social and emotional competencies (as defined by the Collaborative for Academic, Social, and Emotional Learning CASEL. 2003) and school belonging, indicated a strong overall effect size (*r* = 0.44) for the association between competencies and belonging (Allen et al. 2017). Further, the social-emotional learning approach suggests that social-emotional competencies would have direct impacts on short-term student outcomes, including positive social behaviors and relationships in educational settings and indirect effects of these competencies may be important for longer term outcomes, including college and career interest and readiness (Weissberg et al. 2015). Though a theorized relation exists between these two constructs, few studies have examined how social competencies are related to youth belonging in formal or informal educational settings.

### The Importance of Belonging for Math and Science Motivation

Mounting empirical evidence suggests that individuals will be more motivated to engage within a specific domain if they feel a sense of belonging and are included within a domain. For instance, positive relations between school belonging and efficacy beliefs about school have been found for diverse American high school students (Faircloth and Hamm 2005). A study of urban, low-income African American and Latino youth with disabilities indicated that school belonging was positively related to academic self-efficacy and school satisfaction (McMahon et al. 2008).

Fewer studies have examined the relation between interest and school belonging. However, belonginess motivation theory would also posit a positive and predictive effect of belonging on interest, such that youth who feel as though they belong in their educational context will be more interested in the content of that context (Baumeister and Leary 1995). Some prior research examining this found that greater class belonging (in a computer science course) predicted greater interest in enrolling in the course for both girls and boys in a sample of ethnically diverse American high school adolescents (Master et al. 2016). Taken together, from theory and empirical evidence, there appears to be positive relation between belonging and academic efficacy and interest, particularly in classrooms settings. What remains unknown is the extent to which these relations exist in out-of-classroom contexts. Namely, the current study examines whether greater belonging to a program at an informal learning site is related to more motivation in math and science in youth.

### The Role of Informal Learning Contexts in the Shaping of Math and Science Motivation

Much research attention has been paid to math and science motivation in traditional classroom settings (Muenks et al. 2018; Savelsbergh et al. 2016; Wegemer and Eccles 2019). However, evidence is accumulating that more focus should be placed on out-of-classroom or informal science learning sites (ISLS) as a space where STEM motivation can be fostered (National Research Council 2009, 2015). Places such as museums, zoos, aquariums, and other ISLS can serve as unique sources of STEM content and as engaging spaces where learning might occur outside of the formal classroom environment (National Research Council 2009). Indeed, evidence points to the importance of experiences in informal settings as research has noted that ISLS have been shown to foster interest and engagement in STEM domains (Schwan et al. 2014).

Much of the research that examines the relations between social factors, like social competencies and belonging, has been conducted in formal learning contexts (Durlak et al. 2011). Research documents, however, that participating in activities and programs in out-of-school settings may be especially important for a number of developmental domains, including social-emotional development (Mahoney et al. 2005). Further, participating in extracurricular, out-of-school activities is associated with better academic and social outcomes, generally (Eccles et al. 2003; Fredricks and Eccles 2008; Lerner 2005). Adolescents have frequent opportunities to engage in out-of-school experiences with STEM, for instance through extracurricular STEM programming (Habig et al. 2016; National Research Council 2009). Many of these out of school experiences may be short in duration; however, some adolescents have the opportunity to engage in more ongoing, long-term informal STEM participation, for instance youth programming at ISLS (Adams et al. 2015). Furthermore, research suggests that informal STEM learning experiences can have lasting effects on STEM motivation; undergraduates who report prior informal science and math experiences also report higher STEM identity, or perceptions of themselves as STEM oriented (Goff et al. 2019).

The role that social factors and competencies play in explaining increased motivation and interest in STEM for youth who participate in long-term STEM informal learning opportunities has yet to be explored. Moreover, informal learning settings may provide a context where youth can develop a sense of belonging if they do not feel that they fit in at school. In fact, research demonstrates that youth often report that they participate in extracurricular programs for the social benefits, including to have fun and make friends (Borden et al. 2005). Thus, the current study considers how social competencies with peers and STEM program belonging are related to motivational beliefs in math and science among adolescents at informal learning sites.

## Current Study

Though prior research has demonstrated relations between social competencies, belongingness, and math and science efficacy and interest there is a dearth of research working to integrate these relations into a comprehensive model or attend to these relations outside of the formal classroom setting. The current study aimed to fill these gaps in the literature through examining the variables of interest within one model and testing direct and indirect effects of the relations. It was hypothesized that a positive direct effect would be observed between social competencies and belonging to STEM program (Hypothesis 1) and between belonging to STEM program and math and science efficacy and interest (Hypothesis 2). Further, an indirect effect of social competencies on math and science efficacy and interest via belonging to STEM program was expected (Hypothesis 3).

## Methods

### Participants

The study sample was comprised of 268 adolescents (179 girls and 89 boys; *M*_age_ = 15.25*, SD* = 1.81 years), recruited from the U.S. (*n* = 147) and the U.K. (*n* = 121). Participants reflected the ethnic-racial diversity of their communities, with 42.5% White British or European American, 25.7% South Asian British or Asian American, 15.7% Afro-Caribbean Black British or African American, 5.6% Bi-racial, and 3.0% other. Lastly, 7.5% of participants chose not to report their ethnicity. It is important to note that there are differences within these ethnic-racial groups both within and across these countries regarding perceptions of academic abilities. However, participants were parsed in this way for the purposes of understanding the descriptive characteristics of the sample. Participants were recruited through youth educator programs at informal STEM learning sites, where they participated in youth educator programs that involved learning about STEM content and communicating this content to the visitors (for instance, by staffing interactive activities). The sites in the U.S. included a zoo (14.2%), a children’s museum in the U.S. (10.4%) and an aquarium (31.7%). The sites in the U.K. comprised of a science and technology museum (14.6%), and a biomedical and cell biology science learning center (26.9%). Although adolescents were recruited from two different countries, cross-cultural differences across the two subsamples were not expected because the programs at each site are all structured to serve adolescents with similar goals, namely to allow adolescents opportunities to both learn about science domains as well as to engage with the public in some way by serving as guides, docents, and educators at the site. However, to control for any unanticipated effect of country, country of origin was included in the model as a control variable. Sites in the U.S. and the U.K. were chosen as there is a rapidly expanding need for the workforce to be equipped with skills and knowledge of STEM in both of these countries (National Science Board, National Science Foundation. 2015; U.K. Commission for Employment and Skills 2015).

### Procedure

Participants were part of a larger study examining STEM interests and motivation which was approved by the Institutional Review Boards at North Carolina State University and the University of Exeter. Families were notified of the study before enrolling in their STEM program and had the opportunity to select to participate in the study or to opt not to participate. Further, all adolescents assented to participation before completing the survey. Participants were compensated with a small electronic gift card to a retail store of their choice for completion of the study survey. All participants completed the survey within the first months of their yearlong commitment of youth program at the informal science learning site. Participants completed the surveys independently on a computer via Qualtrics.

### Measures

#### Social competencies

An adapted version of Marsh et al. (1983) measure of self-concept of peer relations and social competencies was used to measure social competencies. The six-item measure assesses the extent to which youth believe they have the skills and competencies to engage with their peers. This measure has been used to measure social competencies in individuals from early childhood through emerging adulthood (Shapiro and Martin 2010). Youth indicated their agreement with each item on a four-point scale (1 = *Not at all true*; 4 = *Very true*). Example items read, “I make friends easily” and “I get along with kids easily.” Mean scores were created, where higher scores indicated higher levels of social competencies. The alpha reliability for the scale was good (α = 0.86).

#### Belonging in STEM program

An adapted version of Mendoza-Denton et al. (2002) Institutional Belonging scale was used to measure belonging in STEM program. The original scale was designed to measure college students’ belonging within their STEM major and their comfort and connection to their professors and classmates. In this adapted version of the scale, items were edited from the context of belonging to a STEM major to belonging to a STEM youth program. The scale consisted of eight items. An example item from the scale reads, “How much do you feel that you fit in within your specific STEM program?” Adolescents rated their agreement with each item on a 10-point scale (e.g., 1 = *Definitely do not fit in*; 10 = *Definitely fit in*). Items were averaged to create a mean score of belonging to the STEM program; higher scores indicated higher levels of belonging. The alpha reliability for the scale suggested very high internal consistency across the items (α = 0.95).

#### Math and science efficacy

An adapted version of the Bandura et al. (2001) measure of academic self-efficacy was used to measure both math and science efficacy. The subscales were comprised of 11 items and these subscales were identical with the exception of the subject domain of each item. Youth indicated their agreement with each item on a four-point scale (1 = *Not at all true*; 7 = *Very true*). An example item from the math subscale reads, “How good would you be at learning something new in math?” An example item from the science subscale reads, “How good are you at science?” Alpha reliabilities for the math and science subscales were adequate (α = 0.80, α = 0.76; respectively).

#### Math and science interest

To measure math and science interest, an adapted version of Eccles’ measure of domain-specific interest was employed (Eccles and Wang 2016). The two scales consisted of five items each and were identical with the exception of the domain label. Youth indicated their agreement with each item on a seven-point scale (e.g., 1 = *Not at all interested*; 7 = *Really interested*). An example item from the math subscale reads, “How interested in math are you right now?” An example item from the science subscale reads, “How much do you enjoy science activities right now?” The alpha reliabilities were good for the math and science subscales (α = 0.90, α = 0.77; respectively).

### Analytic Strategy

Descriptive statistics were calculated first to gain a better understanding of characteristics of the sample. A path model was then estimated to examine relations between (a) social competencies and belonging, (b) belonging and math and science efficacy and interest, and (c) the potential indirect relations of social competencies and math and science interest via belonging. An alternative model was also tested where all study outcomes were entered into the model as the most endogenous predictors, followed by belonging in the STEM program, and social competencies. All analyses were conducted using M*plus* Version 8, with full information maximum likelihood estimation (FIML) used to account for missing data (Muthén and Muthén 1998–2019). FIML is a method which handles missing-at-random data by incorporating missing data patterns in the model estimation without listwise deleting incomplete cases (Yuan and Bentler 2000). Simulation studies suggest that FIML is robust under conditions of 50% or more missing data (Enders 2010) and all measures in the current study had missingness levels below this amount. To assess model fit, five goodness-of-fit indices were used: chi-square test of model fit, comparative fit index (CFI), Tucker–Lewis index (TLI), standardized root-mean-square residual (SRMR), and the root mean square error of approximation (RMSEA). Models with a non-significant chi-square test of model fit, a CFI and TLI at or above 0.95, and an SRMR and RMSEA at or below 0.08 were considered acceptable fitting models (Hu and Bentler 1999).

## Results

### Descriptive Analyses

Variable means, standard deviations, and zero-order correlations for all study variables can be found in Table 1. Sample means of math efficacy, science efficacy, and science interest were relatively high, ~1.5 points above the scale midpoints, indicating high levels of efficacy in math and science, and in science interest. Social competencies and math interest were both just above the scale midpoint by ~0.5 point, suggesting youth report relatively average level social competence and math interest. Finally, mean-level belonging to the STEM program was well above the scale midpoint (2.5 points higher than the midpoint of 5.5). Thus, youth in the sample on average felt a strong belonging to their STEM program. Turning to bivariate correlations, a significant positive relation was observed between social competencies and belonging in their STEM program (*r* = 0.43, *p* < 0.01), and belonging in their STEM program and all four of the study outcomes (*r*s > 0.17; *p*s < 0.05).

**Table 1:** Means, Sample Sizes, and Zero-Order Correlations for Key Study Variables (*N* = 268)

	*M* (SD)	*n*	1	2	3	4	5	6
1. Social Competencies	2.94 (0.49)	182	–					
2. Belonging in STEM Program	8.14 (1.52)	149	0.43**	–				
3. Math Efficacy	5.27 (0.91)	256	0.16*	0.20*	–			
4. Science Efficacy	5.97 (0.67)	255	0.07	0.24**	0.30**	–		
5. Math Interest	4.31 (1.47)	182	0.11	0.17*	0.66**	−0.01	–	
6. Science Interest	5.49 (0.96)	183	0.18*	0.31**	0.15	0.53**	0.10	–

*p < 0.05, **p < 0.01

### Path Analyses

**Fig. 1:**
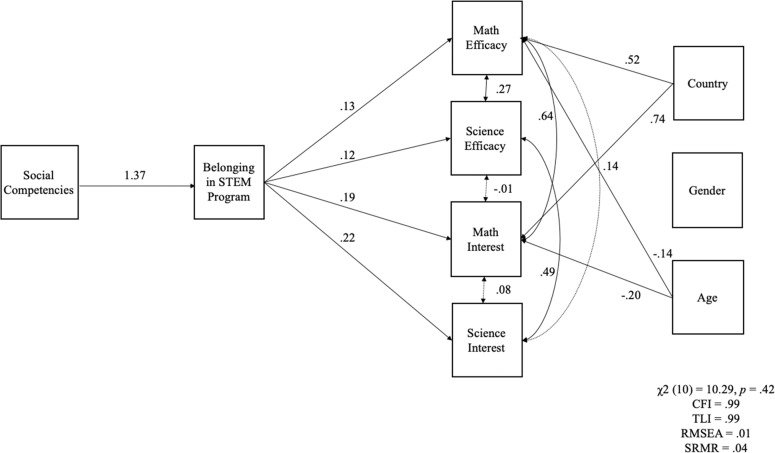
Structural equation model depicting the direct effects of social competencies on belonging in STEM program and belonging in STEM program on math efficacy, science efficacy, math interest, and science interest. Regression weights for unidirectional pathways are unstandardized. Bidirectional pathways are standardized and can be interpreted as correlations. Solid lines represent paths that were significant (*p* < 0.05) and the dashed lines represent paths that were not significant (*p* > 0.05). Only significant paths from control variables were drawn for ease of interpretation

Study hypotheses were tested using a path model (see Fig. 1 for a diagram of the model and model estimates). Belongingness in STEM program was regressed on social competencies. Math efficacy, science efficacy, math interest, and science interest were regressed on belongingness in STEM program. Country of origin (U.S. or U.K.), age, and gender were included in the model as control variables and regressed on the four study outcomes. Given longstanding ethnic/racialized experiences in STEM contexts (e.g., Vakil and Ayers 2019), ethnic racial majority/minority status was included in the model. The ethnic/racial majority for both countries were White (European American or White British), thus a dummy-coded variable was created where White adolescents were considered ethnic majority and all other ethnic-racial groups were considered ethnic minority. However, the inclusion of this control resulted in a significant decrement in model fit and was not a significant predictor of any of the study outcomes; thus, this control variable was not included in the final model. Fit indices for the final model suggested the data fit the model well: *χ*^2^(10) = 10.29, *p* = 0.42; CFI = 0.99; TLI = 0.99; RMSEA = 0.01, [CI = 0.00, 0.07]; SRMR = 0.04. As hypothesized, a positive direct effect of social competencies was observed on belongingness in the STEM program. Further, positive direct effects of belongingness in the STEM program were observed on all four study outcomes. Likewise, there were indirect effects of social competencies on math efficacy, science efficacy, math interest, and science interest via belongness in the STEM program. Results supported the study hypotheses, as all indirect effects were positive and significant for math and science efficacy (*b* = 0.18; *p* = 0.02; *b* = 0.17, *p* = 0.002, respectively) and interest (*b* = 0.6; *p* = 0.02; *b* = 0.31, *p* < 0.001, respectively). In conclusion, results supported each of the three study hypotheses.

## Discussion

Prior research has consistently documented the relations of social competencies, belonging, and motivation outcomes. However, little effort has been made to integrate these social factors into a single model to provide a more comprehensive understanding of the effects of these factors on motivation. Further, to date, research has not explored these relations in contexts outside of formal classroom settings. The current study addresses these gaps by providing a theoretically based structural model of the relations between social competencies, belongingness to STEM programming in an informal learning site, and math and science interest and efficacy. Results confirmed that the more social competency adolescents reported, the more adolescents felt they belonged to their STEM program. Moreover, belonging positively predicted math and science motivation. Finally, there was a positive indirect effect of social competencies on the math and science motivation via belonging.

### The Relations and Roles of Social Competencies and Belonging with Math and Science Motivation

In line with the social-emotional learning perspective, a positive relation between social competencies and belonging was observed. This result indicates that youth with a greater understanding of how to navigate and engage in social relationships with peers report greater feelings of belonging in their informal STEM program. Prior research has shown that social competencies are positively related to, and even predict, feelings of belonging in the school context (Allen et al. 2017). However, no studies have examined this relation for youth involved in programs at ISLS. Documenting the importance of social competencies for belonging to programs in informal settings is an important contribution, as informal settings may provide youth with new peer groups and opportunities to explore their identity and interests in a different setting than formal learning environments. Thus, these results extend knowledge beyond formal learning contexts by pointing to the importance of social competencies in the likely facilitation of belonging in informal learning contexts. The results from the current study also provide support for. The results from the current study provide support for the importance of core social and emotional competencies in alignment with strong focus on relationship skills as posited by the Collaborative for Academic, Social, and Emotional Learning (CASEL) which argues for the importance of explicit social emotional learning for children and adolescents (Osher et al. 2016).

Supporting belongingness motivation theory (Baumeister and Leary 1995), positive relations were observed between belonging and math and science efficacy and interest. Adolescents who reported greater feelings of belonging were likely to report greater math and science efficacy and interest. Only a handful of prior studies have examined relations between adolescents’ academic belonging and efficacy or interest and these studies have exclusively examined belongingness in the context of formal learning contexts (e.g., Faircloth and Hamm 2005; McMahon et al. 2008).

The results from this study provide the field with one of the first domain-specific assessments of the relations between belonging and efficacy and math and science interest in informal STEM learning contexts. With continued interest and calls from governmental agencies to understand ways to enhance STEM motivation in youth (National Science Board, National Science Foundation. 2019), domain specific assessments of these relations can inform future work targeting which social aspects are likely important in shaping STEM motivation. The findings of the current study indicate that feelings of belonging are likely an important factor in the shaping of math and science motivation in adolescent youth. Finally, a primary goal of the study was to test a structural model. The results supported the theorized structure that social competencies are directly associated with belonging but also indirectly associated with math and science efficacy and interest by way of belonging. Thus, these finding provide preliminary evidence for the structure of social constructs that are known to be important and influential in the maintenance and enhancement of math and science motivation among adolescents.

In sum, results from this study contribute to developmental research by providing evidence of the structure of the relations between important social factors that can enhance math and science motivation in learning contexts outside of the traditional classroom. Though these relations have been studied in piecewise examinations in classroom settings. This study is among the first to examine these relations in a comprehensive model and in an informal learning context. Social dynamics can vary from formal and informal learning contexts, thus understanding which social factors are important in out-of-school contexts, as they may or may not be the same as those observed in formal classroom contexts. The results from the current study support theoretical notions and other prior empirical evidence that these social factors are important to consider in adolescents’ math and science motivation in informal learning contexts.

### Implications for Practice

The results from the current study point to the importance of incorporating social-emotional learning practices (for instance, activities to build interpersonal skills, interventions to develop self-regulation and emotional development, or programming to foster youth well-being) into informal out-of-school programming to ensure that participants have the opportunity to connect with and build relationships with their peers and their educators in the program. Benefits of incorporating social-emotional learning in formal learning contexts are well documented (e.g., Greenberg et al. 2017; Taylor et al. 2017); however less is known about how often and in what ways social-emotional learning approaches, particularly those that advance social competencies, are incorporated into youth programming informal learning contexts (Durlak et al. 2010). In addition to attention to education around STEM domains, STEM programs that include opportunities for participants to build their social skills, to form friendships and to connect interpersonally with others will likely be especially successful. Fostering these skills in ISLS are particularly important for adolescent youth as friendships and social group dynamics become increasingly salient and influential on a host of different outcomes, including academic, during adolescence (Brown and Larson 2009).

### Limitations and Future Directions

The current study provides the field with a theoretically informed model as to how social competencies are related to math and science motivation via feelings of belonging in ISLS. However, the study does have some limitations. First, the data are cross-sectional, meaning causation cannot be determined. Thus, tests of directionality are not possible with the data. Another limitation is the use of self-report data. Research has demonstrated that self-reported measures may have method bias, as self-report often acts as a primary source of measurement error (Podsakoff et al. 2003). Future research should aim to extend this work over time and by using observational or behavioral measures, and measures from other reporters, when examining the relations outlined in the study. For example, employing parent, peer or teacher reports of social competencies may be helpful.

Finally, it is important to consider the generalizability of these results. The data were collected from subjects in two Western, developed nations in informal learning contexts. Future research might consider how these social factors might be different in the motivational effect on STEM motivation across various cultures and in formal (classroom) learning contexts. Indeed, within Eastern cultures (Indonesian and Korean) variability has been observed in relations between social factors (parent support) and STEM career motivation (Shin et al. 2018).

## Conclusion

With research demonstrating consistent declines in STEM motivation, it is critical to understand what factors are important in shaping adolescents’ STEM motivation as they prepare to choose their next career steps. Further, it is important to consider these relations in contexts other than the school or classroom as learning occurs in a host of other contexts (Young et al. 2017). To date, little research has examined how social factors that are becoming increasingly important in adolescence could shape adolescent STEM motivation in informal learning contexts. The current study addresses this gap in the literature. The results from this study support the social-emotional learning approach and belongingness motivation theory, as relations between social factors and math and science efficacy and interest were observed. Further, the results underscore the importance of considering how math and science motivation can be impacted in informal learning contexts. The findings provide insight into best practices for development of youth STEM programs, suggesting that programs should attend not only to STEM content, but also to community-building, belonging and fostering the development of key relationships skills in participants.
